# Two faces of lupus nephritis? Questions

**DOI:** 10.1007/s00467-021-04958-4

**Published:** 2021-03-26

**Authors:** Şeyda Doğantan, Neslihan Günay, Sema Nur Taşkın, Aynur Gencer Balaban, Ayşenur Paç Kısaarslan, Sibel Yel, Hülya Akgün, İsmail Dursun, Muammer Hakan Poyrazoğlu

**Affiliations:** 1 Department of Pediatric Rheumatology, Erciyes University Medical Faculty, Kayseri, Turkey; 2 Department of Pediatric Nephrology, Erciyes University Medical Faculty, Kayseri, Turkey; 3 Pathology Department, Erciyes University Medical Faculty, Kayseri, Turkey

**Keywords:** Child, Hemolytic anemia, Polyarthralgia, Proteinuria, Glomerulonephritis, Systemic lupus erythematosus

## Case

An 11-year-old girl was admitted with hemolytic anemia and polyarthralgia that had persisted for 6 months. Her family history was remarkable for Sjogren’s syndrome. On admission, she had a pallor appearance and arthralgia on the ankles and knees.

Laboratory findings were as follows: hemoglobin 4.7 g/dL, white blood cell count 8000/mm^3^, platelets 210.000/mm^3^, reticulocyte ratio 7.9%, BUN 16 mg/dL, creatinine 0.41 mg/dl, LDH 576 U/L, ANA 1:1000, anti-ds-DNA negative, ACA positive, anti-beta2 glycoprotein antibody positive, serum C3 and 4 normal, direct globulin positive, lupus anticoagulant ratio 1.27 (1+), p-ANCA and c-ANCA negative. A 24-h urine collection showed nephrotic range proteinuria of 1632 mg/m^2^/day.

**Fig. 1:**
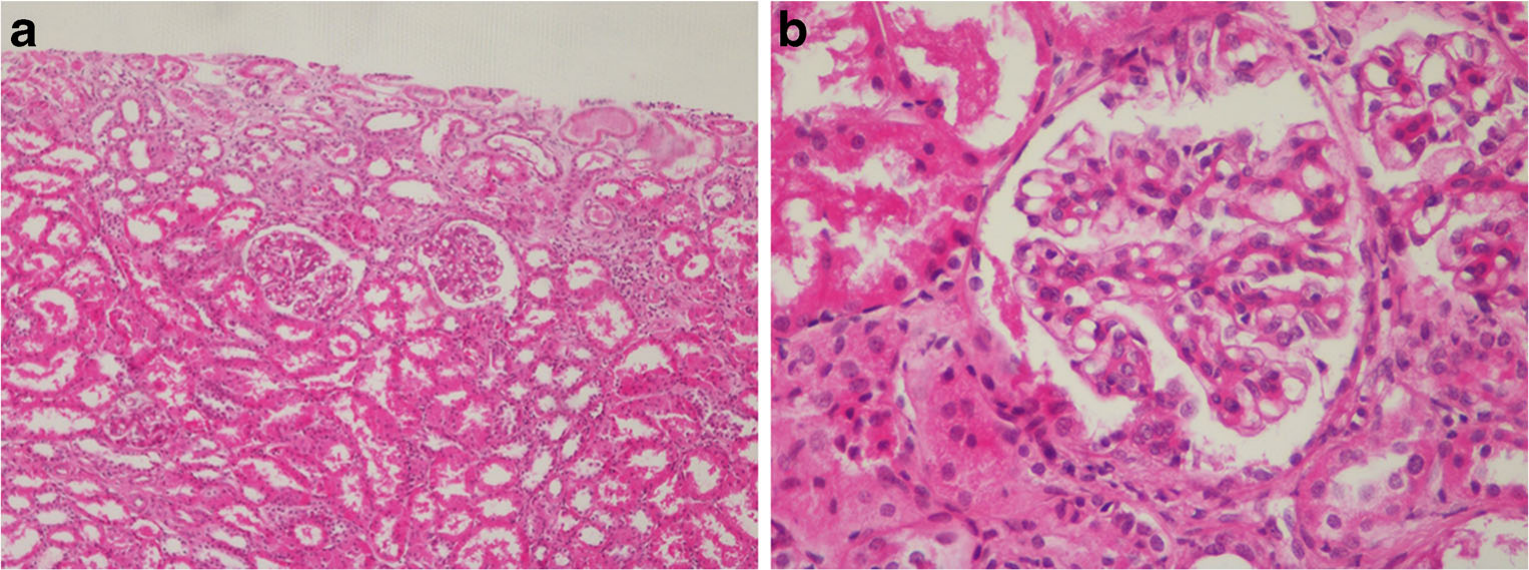
Pathological findings from kidney biopsy. Light microscopy revealed mild mesangial cell proliferation (**a** hematoxylin eosin, ×100 and **b** hematoxylin eosin, ×400)

Kidney ultrasound revealed increased renal parenchymal echogenicity. Kidney biopsy was performed and demonstrated mild mesangial cell proliferation without any immunofluorescence staining for IgG, IgM, IgA, C3, C1q, kappa, or lambda light chains (Fig. 1a, b).

Based on the clinical and laboratory findings, she was diagnosed with systemic lupus erythematosus (SLE) and treated with steroids, mycophenolate mofetil (MMF), and hydroxychloroquine since kidney biopsy was compatible with mesangial proliferative glomerulonephritis.

### Questions

1. What is your diagnosis based on the renal histopathologic findings?
2. What is the best tool for diagnosis?
3. What is the possible underlying mechanism of kidney involvement?
4. What are the treatment options for the patient?

